# Combining Entropy Measures for Anomaly Detection

**DOI:** 10.3390/e20090698

**Published:** 2018-09-12

**Authors:** Alberto Muñoz, Nicolás Hernández, Javier M. Moguerza, Gabriel Martos

**Affiliations:** 1 Department of Statistics, Universidad Carlos III de Madrid, 28903 Getafe, Madrid, Spain; 2 Department of Computer Science and Statistics, University Rey Juan Carlos, 28933 Móstoles, Madrid, Spain; 3 Department of Mathematics and Statistics, Universidad Torcuato Di Tella and CONICET, Buenos Aires C1428BCW, Argentina

**Keywords:** entropy kernel, kernel combination, Karcher mean, anomaly detection, functional data

## Abstract

The combination of different sources of information is a problem that arises in several situations, for instance, when data are analysed using different similarity measures. Often, each source of information is given as a similarity, distance, or a kernel matrix. In this paper, we propose a new class of methods which consists of producing, for anomaly detection purposes, a single Mercer kernel (that acts as a similarity measure) from a set of local entropy kernels and, at the same time, avoids the task of model selection. This kernel is used to build an embedding of data in a variety that will allow the use of a (modified) one-class Support Vector Machine to detect outliers. We study several information combination schemes and their limiting behaviour when the data sample size increases within an Information Geometry context. In particular, we study the variety of the given positive definite kernel matrices to obtain the desired kernel combination as belonging to that variety. The proposed methodology has been evaluated on several real and artificial problems.

## 1. Introduction

Usual Data Mining tasks, such as classification, regression and anomaly detection, are heavily dependent on the geometry of the underlying data space. Kernel Methods, such as Support Vector Machines (SVM), provide the control on the data space geometry through the use of a Mercer kernel function [1,2]. Such functions, defined in the next section, induce embeddings of the data in feature spaces where Mercer kernels act as inner products. The choice of the appropriate kernel, including its parameters, is a particular case of model selection problems.

For instance, when working with SVM, a delicate parameterization is needed; otherwise, solutions might be suboptimal. In other words, the choice of a suitable kernel function and its parameters will affect both the geometry of the data embedding and the success of the algorithms [3,4]. A typical way to proceed is by means of cross-validation procedures [5]. However, these parameter calibration strategies, although intuitive and simple from an applied point of view, have some important drawbacks. In particular, their computational burden is of practical relevance when implementing cross–validation strategies in problems that involve calibrating a medium to large amount of parameters. An appealing alternative to model selection when working with SVM is to combine or merge different kernel functions into a single kernel [6,7].

Functional data [8] present the particularity of being intrinsically infinite dimensional. This peculiarity implies that classical procedures for multivariate data must be adapted or redesigned to cope with functional data. The statistical distribution of data is a basic element to afford outlier detection problems. Entropies are natural functions to use in anomaly detection problems given that any definition of entropy should produce large values for scattered distributions and small values for concentrated distributions. In addition, statistical distributions are a particular case of functional data and in this way entropy comes then into play in this context.

In this paper, we present an alternative proposal to solving anomaly detection problems that avoids the selection of kernel hyperparameters. A novelty of this work is that the methodology is developed to deal with functional data. We will explore several kernel combination techniques, including some methods from Information Geometry that respect the geometry of the manifold that contains the Gram matrices associated with the Mercer kernels involved.

The paper is organized as follows: Section 2 describes the functional data analysis methods used to produce the data representations from kernels, as well as the minimum entropy method used in this paper for anomaly detection. Section 3 develops several methods to obtain kernel combinations for the task of outlier detection. Section 4 illustrates the theory with simulations and examples; and Section 5 concludes the work.

## 2. Reproducing Kernel Hilbert Spaces for Multivariate and Functional Data

Let *X* be the “space” where the data live (a compact metric space). A Mercer kernel is a function K:X×X→R symmetric, continuous and such that, for all finite sets $S=\{x_{1},\;\ldots ,\;x_{n}\}\subset X$, the matrix whose entries are $K{\left(x_{i},x_{j}\right)}_{i,j\in \{1,\;\ldots ,\;n\}}$ is positive semidefinite. Often, we will use the term “kernel function” when referring to a Mercer kernel. Kernel functions admit expansions of the type $K\left(x,z\right)=\sum_{i}\phi {\left(x\right)}^{T}\phi \left(z\right)$ for some $\phi :X⟶R^{d}$, where *d* is usually large. In particular, ϕ(X) is some manifold embedded in $R^{d}$ [9]. For x∈X, denote $K_{x}$ the function $K_{x}:X\to R$ given by $K_{x}\left(z\right)=K\left(x,z\right)$. There exists a unique Hilbert space $H_{K}$ of functions on *X* made up of the span of the set $\left\{K_{x}|x\in X\right\}$, such that for all $f\in H_{K}$ and x∈X, $f\left(x\right)={\langle K_{x},f\rangle }_{H_{K}}$. The Hilbert space $H_{K}$ is said to be a Reproducing Kernel Hilbert Space (RKHS) [10]. Next, we describe the use of RKHS for data analysis, differentiating between the multivariate and functional cases.

In the multivariate case, we consider data sets $S=\{x_{1},\ldots ,x_{n}\}\subset X,$ where *X* is a compact subset of $R^{D}$. Consider the RKHS $H_{K}$ and the linear integral operator $L_{K}$ defined by $L_{K}\left(f\right)=\int_{X}K\left(\cdot ,s\right)f\left(s\right)ds$. Since *X* is compact and K continuous, $L_{K}$ has a countable sequence of eigenvalues $\left\{\lambda_{j}\right\}$ and eigenfunctions $\left\{\phi_{j}\right\}$, and *K* can be expressed as $K\left(x,y\right)=\sum_{j}\lambda_{j}\phi_{j}\left(x\right)\phi_{j}\left(y\right)$, where the convergence is absolute and uniform (Mercer’s theorem).

Consider the Gram matrix $K_{S}=K\left(x_{i},x_{j}\right)$, i,j∈{1,…,n}. This matrix is real, symmetric and positive definite (by definition of *K*) and $K_{S}\left(x_{i},x_{j}\right)=\phi {\left(x_{i}\right)}^{T}\phi \left(x_{j}\right)$, where $\phi \left(x_{i}\right)={\left(\sqrt{\lambda_{j}}\phi_{j}\left(x_{i}\right)\right)}_{j}$ is the mapping $\phi :X\to R^{d}$. Straightforwardly, *K* is the standard scalar product in $R^{d}$. Thus, the use of *K* induces both a data transformation and a metric on the original data given by:

(1) $$d_{K}^{2}\left(x_{i},x_{j}\right)={∥\phi \left(x_{i}\right)-\phi \left(x_{j}\right)∥}^{2}=\phi {\left(x_{i}\right)}^{T}\phi \left(x_{i}\right)+\phi \left(x_{j}\right)\phi \left(x_{j}\right)-2\phi \left(x_{i}\right)\phi \left(x_{i}\right)=K\left(x_{i},x_{i}\right)+K\left(x_{j},x_{j}\right)-2K\left(x_{i},x_{j}\right)$$

Equation (1) shows that the choice of the kernel *K* determines the geometry of the data set after the transformation X→ϕ(X).

Now, we consider the functional data case, that is, the case where data are functions or, by generalization, infinite dimensional objects (such as images, for instance). Let (Ω,F,P) be a probability space, where F is the σ-algebra in Ω and *P* a σ-finite measure. We consider random elements (functions) X(ω,t):Ω×T→R in a metric space (T,τ), where T⊂R is compact and we assume X(ω,·) to be continuous functions. We consider kernels $K_{X}\left(s,t\right)=K\left(X\left(\omega ,s\right),X\left(\omega ,t\right)\right)$ (the classical choice is $K_{X}\left(s,t\right)=E\left(X\left(\omega ,s\right)X\left(\omega ,t\right)\right)$). Then, there exists a basis ${\left\{e_{i}\right\}}_{i\geq 1}$ of C(T) such that for all t∈T

(2) $$X\left(\omega ,t\right)=\sum_{i=1}^{\infty }\xi_{i}\left(\omega \right)e_{i}\left(t\right),$$

for appropriate coefficients, where the $e_{i}$ are the eigenfunctions associated with the integral operator of $K_{X}\left(s,t\right)$.

In real data analysis, we do not have theoretical random paths, or functional data described by mathematical equations, but finite samples from such processes. For instance, if we are considering normal distributions as the object of analysis, we will not know the vectors of means and real covariance matrices(μ and Σ), but a sample $X=\left\{x_{i}\right\}\in R^{n}$ from which we will estimate the covariance matrix $S=\frac{1}{n}XX^{T}$. In the case of functions, *X* will be a compact space or manifold in an Euclidean space, Y=R, and there will be available sample curves $f_{n}$ identified with data sets ${\left\{\left(x_{i},y_{i}\right)\in X\times Y\right\}}_{i=1}^{n}$. Let K:X×X→R a Mercer Kernel and $H_{K}$ its associated RKHS. Then, the coefficients in Equation (2) can be approximated by solving the following optimization problem [11]:

(3) $$\min_{f\in H_{K}}\frac{1}{n}\sum_{i=1}^{n}{\left(f\left(x_{i}\right)-y_{i}\right)}^{2}+\gamma {∥f∥}_{K}^{2},$$

where γ>0 and ${∥f∥}_{K}^{2}$ represents the norm of the function *f* in $H_{K}$. The solution, that constitutes an example of Equation (2), is given by $f^{*}\left(x\right)=\sum_{j}{\hat{\lambda }}_{j}\phi_{j}\left(x\right)$, where the ${\hat{\lambda }}_{j}$ are the weights of the projection of the function corresponding to the sample $\left\{\left(x_{i},y_{i}\right)\right\}$ onto the function space generated by the eigenvalues of $L_{K}$.

Next, we use local entropies for anomaly detection through kernel combinations. For this preliminary work, we explore linear combinations and Karcher means, to validate the intuition that the use of a more natural mean than the arithmetic mean will produce better practical results, as far as positive definite matrices are involved.

### 2.1. Local Entropy Kernels

In order to link the metric induced by the kernel function and the underlying (empirical) density in the data, we propose *local entropy kernels*. Consider a measurable cover on (Ω,F,P)—the probability space where the random element of interest *X* is defined—say ${\left\{\Omega_{i}\right\}}_{i\geq 1}$, where ${⋃}_{i\geq 1}\Omega_{i}=\Omega$ and $\Omega_{i}\cap \Omega_{j}=\emptyset$ for any i≠j; we can define the α-Entropy [12] of *X* as follows:

(4) $$H_{\alpha }\left(X\right)=\frac{1}{1-\alpha }\sum_{i\geq 1}P\left(\Omega_{i}\right)\log\left(P{\left(\Omega_{i}\right)}^{\alpha -1}\right),\;\mathrm{for}\;\alpha \geq 0\;\mathrm{and}\;\alpha \neq 1.$$

The parameter α defines to which entropy inside the family of α entropies we are referring to. For instance, when α=0, then $H_{\alpha }$ is the Hartley entropy, when α→1 then $H_{\alpha }$ converges to the Shannon entropy and when α→∞ then $H_{\alpha }$ converges to the Min-entropy measure. Let $S_{\Omega }$ be the collection of finite partitions of Ω, for any subset $A={⋃}_{i=1}^{n}A_{i}\in S_{\Omega }$, the entropy of *A* can be computed as follows:

(5) $$H_{\alpha }\left(A\right)=\frac{1}{1-\alpha }\sum_{i=1}^{n}P\left(A_{i}\right)\log\left(P{\left(A_{i}\right)}^{\alpha -1}\right).$$

This paves the way to define the Δ-local entropy [13] corresponding to any subset $\Delta \in F_{\Omega }$ as follows

(6) $$h_{\alpha }\left(\Delta \right)=\underset{\tilde{\Delta }\in S_{\Omega }}{\inf}H_{\alpha }\left(\tilde{\Delta }\right),\;\mathrm{such}\;\mathrm{that}\;\Delta \subset \tilde{\Delta }.$$

Let $\left(X_{1},\;\ldots ,\;X_{n}\right)$ be a random sample drawn i.i.d. from *P*, we would like to compute the local entropies of the corresponding random sets $\Delta_{1},\;\ldots ,\;\Delta_{n}$, where $\Delta_{i}=\Omega ⋂B\left(X_{i}^{-1}\left(\omega \right),r\right)$ and $B(X_{i}^{-1}\left(\omega \right),r)\in \Omega$ is the open ball with center in ω and a (data driven) small radius *r*. In practice, given a sample $S_{n}=\left(x_{1},\;\ldots ,\;x_{n}\right)$, we compute the local entropy using the estimator ${\hat{h}}_{\alpha }\left(\Delta_{i}\right)={\bar{d}}_{k}\left(x_{i},S_{n}\right)/\left(1-\alpha \right)$, where ${\bar{d}}_{k}\left(x_{i},S_{n}\right)$ is the average distance from $x_{i}$ to its $k^{th}$-nearest neighbour. Notice that the locality parameter *k* in ${\bar{d}}_{k}\left(x,S_{n}\right)$, which represents the number of neighbours that we take into account to approximate the local entropy around *x*, is related to *r* in $\Delta_{x}=\Omega ⋂B\left(x^{-1}\left(\omega \right),r\right)$. We then consider $\varphi \left(x\right)={\hat{h}}_{\alpha }\left(\Delta_{x}\right)$, with α=0, so to define the local entropy kernel as

(7) $$K\left(x,y\right)=\varphi {\left(x\right)}^{T}\varphi \left(y\right).$$

In the next section, we discuss how to avoid model selection problems. To this aim, a set of local entropy kernels is initially estimated from the data. Then, we estimate an average local entropy kernel that takes into account the particular geometry of the space of positive definite matrices. In this way, we obtain a unique low dimensional data representation, from which outliers are detected. This approach does not include neither a model selection step nor a parameter estimation procedure.

## 3. Kernel Combination for Anomaly Detection

Consider a data sample $S_{n}=\left\{x_{1},\ldots ,x_{n}\right\}\subset X$, where the $x_{i}$ can be multivariate or functional data, and consider a set of *m* Mercer kernels (or matrices) $K_{1}^{e},\ldots ,K_{m}^{e}$, that induce *m* different data embeddings $\phi_{j}:X\to R^{d_{j}}$, where $K_{j}^{e}\left(x,y\right)=\phi_{j}{\left(x\right)}^{T}\phi_{j}\left(y\right)$. As stated in Equation (1), each of the kernels induces a kernel distance $d_{K_{j}}$ on the original data space *X*, corresponding to the Euclidean distance on the manifold $Z_{j}=\phi_{j}\left(X\right)$.

Next, we define a new set of transformations, suitable for anomaly detection, in line with the theory of Section 2.1 by:

(8) $$\varphi_{j}\left(x\right)=d_{K_{j}}\left(\phi_{j}\left(x\right),\phi_{j}\left(S_{n}\right)\right).$$

The corresponding kernels suitable for outlier detection are

(9) $$K_{j}\left(x,y\right)=\varphi_{j}{\left(x\right)}^{T}\varphi_{j}\left(y\right).$$

Now, kernel functions are positive definite type functions, i.e., the empirical kernel matrix *K*—obtained via the evaluation of the kernel function into the set of *n* training points—belongs to the cone of symmetric positive semidefinite matrices $P:=\{K\in R^{n\times n}|K=K^{T},K⪰0\}$. Let $K_{1},\;\ldots ,\;K_{m}$ be the empirical kernel matrices defined in Equation (9), all of them in P, and let ${\left(w_{1},\;\ldots ,\;w_{m}\right)}^{T}$ be a suitable non-negative vector of combination parameters, then define the “fusion” kernel K

$$K\left(w_{1},\;\ldots ,\;w_{m}\right):=w_{1}K_{1}+,\;\ldots ,\;+w_{m}K_{m}⪰0.$$

In the context of SVM classification problems, the goal is to find the parameters $w_{1},\;\ldots ,\;w_{m}$ that maximize the optimal margin. Instead, in anomaly detection cases, the goal is to estimate the parameters $w_{1},\;\ldots ,\;w_{m}$ that produce a suitable data representation. This is achieved when the regular data within the sample –represented in the coordinate space provided by the fusion kernel K–have a reduced entropy or equivalently is scarcely scattered and those observations that are atypical in the sample are projected in distant regions from that of the regular data.

Next we consider three particular combination schemes. The first is rather straightforward, the second proposes the mean in the manifold that contains the kernels, and the third is a weighting scheme that assigns the weights according to the use of appropriate choices of entropy functions.

**Definition** **1** (Multivariate sparsity measures). *Consider m different sparsity measures $\phi_{1},\;\ldots ,\;\phi_{m}$ and let $K_{1},\;\ldots ,\;K_{m}$ be the corresponding set of Mercer kernels, where $K_{i}\left(x,y\right)=\phi_{i}^{T}\left(x\right)\phi_{i}\left(y\right)$. We define a multivariate concentration measure by $\Phi =\left(\phi_{1},\;\ldots ,\;\phi_{m}\right):X\to R^{m}$.*

The corresponding kernel, evaluated at the sample S, will be

(10) $$\begin{array}{ccc} K_{\Phi }\left(x_{i},x_{j}\right)=\Phi {\left(x_{i}\right)}^{T}\Phi \left(x_{j}\right) & = & {\left(\phi_{1}\left(x_{i}\right),\ldots ,\phi_{m}\left(x_{i}\right)\right)}^{T}\left(\phi_{1}\left(x_{j}\right),\ldots ,\phi_{m}\left(x_{j}\right)\right)=\sum_{i=1}^{m}\phi_{i}^{T}\left(x_{i}\right)\phi_{i}\left(x_{j}\right)=\sum_{i=1}^{m}K_{i}\left(x_{i},x_{j}\right) \end{array}$$

Thus, the kernel corresponding to a multivariate sparsity measure $\Phi =(\phi_{1},\;\ldots ,\;\phi_{m})$ is the sum of the univariate kernels $K_{i}$ associated with the $\phi_{i}$. This fact allows us to interpret linear combination of kernels $\sum w_{i}K_{i}$ as coming from (weighted) multivariate sparsity measures.

### 3.1. Entropy Weighting

**Definition** **2** (K-entropy of a data set). *Consider a Mercer kernel K acting on a space X, a sample data set $S_{n}$ and the corresponding transformation $\phi :X\to R^{d}$ induced by K, where $K\left(x,y\right)=\phi {\left(x\right)}^{T}\phi \left(y\right)$. The* ***K-entropy*** *of $S_{n}$ is defined by:*

(11) $$E_{K}\left(S_{n}\right)=\sum_{i=1}^{n}\sum_{j=1}^{n}|K\left(x_{i},x_{j}\right)|=\sum_{i=1}^{n}\sum_{j=1}^{n}\left|\phi {\left(x_{i}\right)}^{T}\phi \left(x_{j}\right)\right|.$$

In the context of outlier detection, consider $K_{1},\;\ldots ,\;K_{m}$, obtained from sparsity measures. From Equation (9), if a point *x* is an outlier, then it will be off the main bulk of data points and, thus, $\varphi_{j}\left(x\right)=d_{K_{j}}\left(\phi_{j}\left(x\right),\phi \left(S_{n}\right)\right)$ will be large and the same will be true for $K_{j}\left(x,x_{i}\right)$ for most $x_{i}\in S_{n}$. As a consequence, $E_{K_{j}}\left(S_{n}\right)$ will tend to be large. On the other hand, and following the same reasoning, if a particular kernel $K_{j}$ induces a representation not suitable for detecting the outliers, then $E_{K_{j}}\left(S_{n}\right)$ will be small. Thus, the measure defined in Equation (11) acts as a true entropy for matrices: If data are very concentrated after the transformation induced by *K*, then the entropy of the data (measured by the) set will be low.

We establish the entropy-weighting scheme by solving the following semidefinite optimization problem:

(12) $$\begin{array}{c} \max_{\alpha_{1},\;\ldots ,\;\lambda_{m}}\sum_{j=1}^{m}\lambda_{j}E_{K_{j}}\left(S_{n}\right)\\ s.t.\sum_{j=1}^{m}\lambda_{j}K_{j}⪰0,\;\sum_{j=1}^{m}\lambda_{j}=1,\;\mathrm{and}\;0\leq \lambda_{j}\leq u_{j}, \end{array}$$

where $u_{j}\in [\left(0,1\right]$ are some positive constants that may be associated with each kernel matrix $K_{j}$. We refer to [14] for a detailed description of the basics of semidefinite programming.

**Theorem** **1.** Consider the previous semidefinite optimization problem. If $K_{1},\;\ldots ,\;K_{m}⪰0$ and $u_{i}=\frac{E_{K_{j}}\left(S_{n}\right)}{\sum_{j}E_{K_{j}}\left(S_{n}\right)}$, then the solution to the optimization problem is given by $\lambda_{j}^{*}=\frac{E_{K_{j}}\left(S_{n}\right)}{\sum_{j}E_{K_{j}}\left(S_{n}\right)}$.

**Proof.**  Given that $\lambda_{j}^{*}=\frac{E_{K_{j}}\left(S_{n}\right)}{\sum_{j}E_{K_{j}}\left(S_{n}\right)}\geq 0$ and $K_{j}⪰0$, the constraint $\sum_{j=1}^{m}\lambda_{j}K_{j}⪰0$ holds. In addition,

$$\sum_{j=1}^{m}\lambda_{j}^{*}=\sum_{j=1}^{m}\frac{E_{K_{j}}\left(S_{n}\right)}{\sum_{j}E_{K_{j}}\left(S_{n}\right)}=1.$$

Since all the $\lambda_{j}^{*}$ reach their upper bound, the theorem holds and the solution is unique. □

Thus, the entropy-weighting scheme will be:

(13) $$K^{*}=\frac{E_{K_{1}}\left(S_{n}\right)}{\sum_{j}E_{K_{j}}\left(S_{n}\right)}K_{1}+\frac{E_{K_{2}}\left(S_{n}\right)}{\sum_{j}E_{K_{j}}\left(S_{n}\right)}K_{2}+\ldots +\frac{E_{K_{m}}\left(S_{n}\right)}{\sum_{j}E_{K_{j}}\left(S_{n}\right)}K_{m}.$$

### 3.2. Karcher Mean

Next, we introduce the Karcher mean [15,16,17] of kernel matrices as an alternative approach to the linear combinations of matrices presented in Section 3.1. The Karcher mean preserves the particular Riemannian manifold in which the kernel matrices lie and constitutes a natural definition for the geometric mean of the matrices.

The set of positive definite square matrices P is a Riemannian manifold, with inner product ${\langle A,B\rangle }_{X}=Tr\left(X^{-1}AX^{-1}B\right)$ on the tangent space to P at the point *X*. The distance between A,B∈P is given by $d_{P}\left(A,B\right)={∥\log\left(A^{-1/2}BA^{-1/2}\right)∥}_{F}$, where ${∥\cdot ∥}_{F}$ is the Frobenius norm, that is ${∥A∥}_{F}=\sqrt{\sum_{i}\sum_{j}a_{ij}^{2}}$. Given $K_{1},\ldots ,K_{m}$ kernel matrices, the Karcher mean, denoted onwards as $\bar{\bar{K}}$, is defined as the minimizer of the function $f\left(X\right)=\sum_{i=1}^{m}d_{P}{\left(X,K_{i}\right)}^{2}$, and it is the unique solution X∈P of the matrix equation $\sum_{i=1}^{m}\log\left(K_{i}^{-1}X\right)=0$.

## 4. Experimental Section

In this section, we illustrate, with the aid of multiple numerical examples and real data sets, the performance of the proposed methodology when the goal is to detect abnormal observations in a sample. We consider a list of several kernel functions, namely: (i)  the Gaussian kernel $K_{G}\left(x_{i},x_{j}\right)=e^{-\sigma ∥x_{i}-x_{j}{∥}^{2}}$ with parameter σ defined in a grid of values ranging in $\sigma \in \{0.1^{3},0.1^{2},0.1,1,10,50,100,500,10^{3}\}$; (ii) the linear kernel $K_{L}\left(x_{i},x_{j}\right)=\langle x_{i},x_{j}\rangle$ and (iii) the second degree polynomial kernel $K_{P}\left(x_{i},x_{j}\right)={\left(\langle x_{i},x_{j}\rangle +1\right)}^{2}$. As it was explained in Section 1, the combination methods proposed can be considered as an alternative to model selection techniques for outlier detection purposes. Therefore, the results obtained are presented jointly with the single kernel methods. Our combination methods are denoted as: (i) the average kernel (*K*), (ii) the kernel constructed using the *Karcher* mean of the single kernel functions ($\bar{\bar{K}}$) and (iii) the minimum entropy linear combination kernel or entropy kernel ($E_{K}$).

For comparison purposes, we consider several alternative approaches for anomaly detection in both the multivariate and the functional data frameworks. In the multivariate case, we consider some alternative well-known techniques in the field of machine learning. These methods are: (i) LOF [18] and (ii) HiCS [19]. In the functional case, we test our proposals against three widely used depth measures: the Modified Band Depth (MBD) [20], the Modal Depth (HMD) [21] and the Random Tukey Depth (RTD) [22]. This depth measures induce an order with respect to the functional data set that can be used to determine which observations (curves) are far from the deepest or central point and can be classified as outliers.

Each database presents a set of regular observations and has been contaminated with abnormal or outlying observations. Let P and N be the amount of outlier and normal data in the sample, respectively, and let TP = True Positive and TN = True Negative be the respective quantities detected by different methods. In Table 1 and Table 2, we report the following average metrics TPR = TP/P (True Positive Rate or sensitivity), TNR = TN/N (True Negative Rate or specificity). For the comparison with other techniques using real data sets, in Table 4 and Table 5, we report the area under the ROC curve (AUC) for each experiment.

### 4.1. Synthetic Data

For the simulated experiment, we consider two synthetic data schemes. The first scheme has been built by generating a synthetic multivariate data set, while, for the second scheme, we have generated a synthetic functional data set.

**Figure 1:**
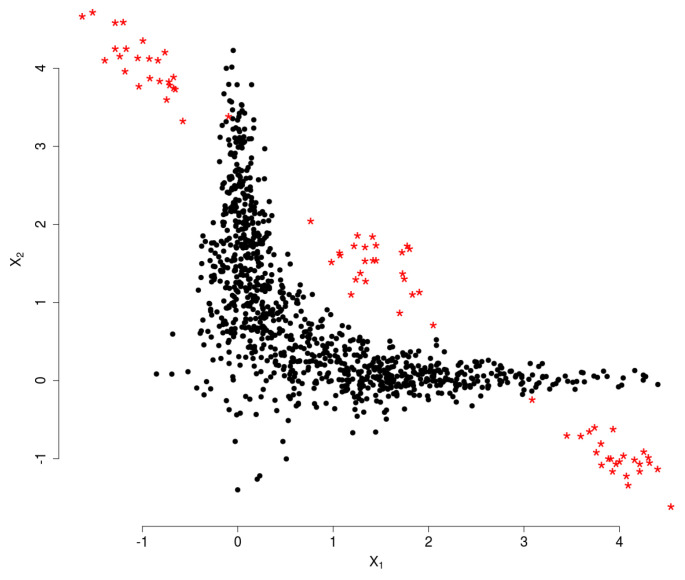
Main data in black (●) and outlying observations in red (*).

**Synthetic multivariate data:** We consider a conditionally normal bivariate distribution model [23] for regular data and outliers were sampled from three different standard Gaussian models. The sample size is n=1000. The data for the experiment, illustrated in Figure 1, was obtained using a Gibbs sampler.

**Figure 2:**
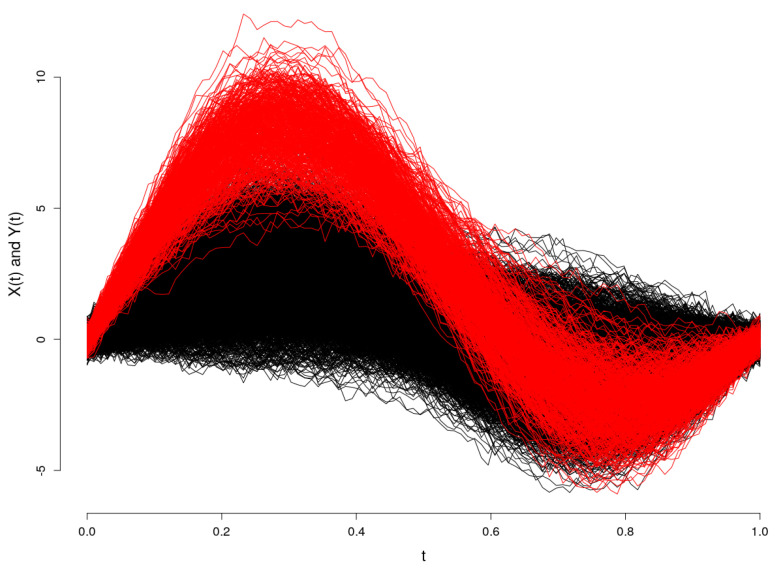
Main data in black (—) and outlying observations in red (—).

**Synthetic functional data:** We consider random samples of Gaussian processes $\left\{x_{1}\left(t\right),\;\ldots ,\;x_{n}\left(t\right)\right\}$, with sizes 4000 and 2000, where a proportion ν=0.1, known a priori, present an atypical pattern, and the remaining n(1−ν) curves are considered the main data. We consider the following generating processes:

$$\begin{array}{ccc} X_{l}\left(t\right) & = & \sum_{j=1}^{2}\xi_{j}\sin\left(j\pi t\right)+\varepsilon_{l}\left(t\right),\;\mathrm{for}\;l=1,\;\ldots ,\;\left(1-\nu \right)n,\\ Y_{l}\left(t\right) & = & \sum_{j=1}^{2}\zeta_{j}\sin\left(j\pi t\right)+\varepsilon_{l}\left(t\right),\;\mathrm{for}\;l=1,\;\ldots ,\;\nu n, \end{array}$$

where t∈[0,1], ε(t) are independent autocorrelated random error functions and $\left(\xi_{1},\xi_{2}\right)$ is a normally-distributed bivariate random variable (NDMRV) with mean $\mu_{\xi }=\left(1,2\right)$ and diagonal co-variance matrix $\Sigma_{\xi }=\mathrm{diag}\left(1,1\right)$. To generate the outliers, we consider $\left(\zeta_{1},\zeta_{2}\right)$ NDMRV with parameters $\mu_{\xi }=\left(4,5\right)$ and $\Sigma_{\zeta }=\Sigma_{\xi }$. The data are plotted in Figure 2.

Table 1 shows the results of the experiment using synthetic multivariate data. Best results are marked using bold enhanced text. It can be observed that the proposed combination methods, namely the mean, the weighted entropy and the Karcher mean perform as well as the best single kernel in terms of the TNR. With respect to the TPR, the best combination method is the one based on the calculation of the Karcher mean.

In Table 2, the results of the experiment using synthetic functional data are presented. In this case, two of the three proposals, the mean and the weighted entropy are always able to perform as well as the best single kernel (the polynomial kernel) in terms of both the TNR and TPR. The method based on the calculation of the Karcher mean obtains good results with respect to the TNR measure.

### 4.2. Real Data

**Figure 3:**
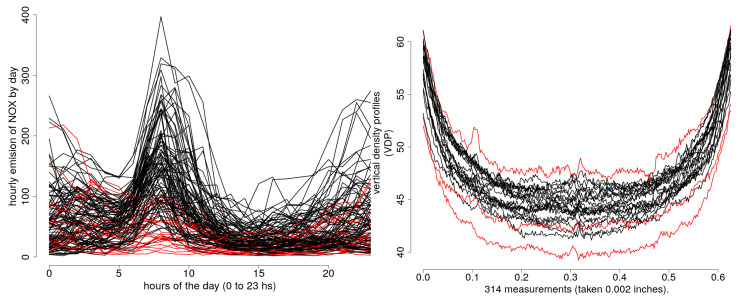
NOX (**left**) and VDP (**right**) functional data sets. The sample of regular curves in black (“—”), and abnormal curves in red (“—”).

Regarding real data, we also differentiate between multivariate and functional data. To test and compare proposals using multivariate data, we consider six databases from the UCI machine learning repository [24] which are available and properly described in [25]. The testing and comparison of our proposals using functional data are carried out over two functional data sets: (i) Poblenou NOx Emissions (NOx). This data set contains the nitrogen oxide ($NO_{x}$) emissions levels measured every hour by a control station in Poblenou in Barcelona (Spain). The data are publicly available in the R-package `fda.usc’ [26]. In the data set, working day $NO_{x}$ emissions, considered as regular data, and weekend day $NO_{x}$ emissions considered as atypical data can be distinguished; (ii) Vertical Density Profiles (VDP). This data set contains 24 curves of Vertical Density Profiles which come from the manufacture of engineered woodboards. Each one consists of 314 measurements taken 0.002 inches (see [27] for further details). In Table 3, we give the details about the sample size, the dimension and the percentage of outlier observations for each of the data sets. The NOx and VDP data sets are illustrated in Figure 3.

Table 4 shows the results of the experiment using real multivariate data. It can be observed that the best overall method in average is the weighted entropy proposal. In particular, this method attains the best results for two of the six data bases (Pima and Cardio), and for the rest of the sets its results are close to the best ones. Although the proposed methodologies seem to perform systematically better than other machine learning approaches, it is not clear, in terms of the AUC, whether for some data bases (Glass, Breast Cancer, Breast Cancer Diagnostic and Pima) the difference is statistically significant.

In Table 5, the results of the experiment using real functional data are presented. For the VDP data set, in terms of the AUC measure, the weighted entropy and the mean proposals perform as well as the best single kernels and the MBD. For the NOx data set, the best overall method is the one based on the calculation of the Karcher mean, followed closely by the MBD approach.

### 4.3. Robustness of the Karcher Mean

**Figure 4:**
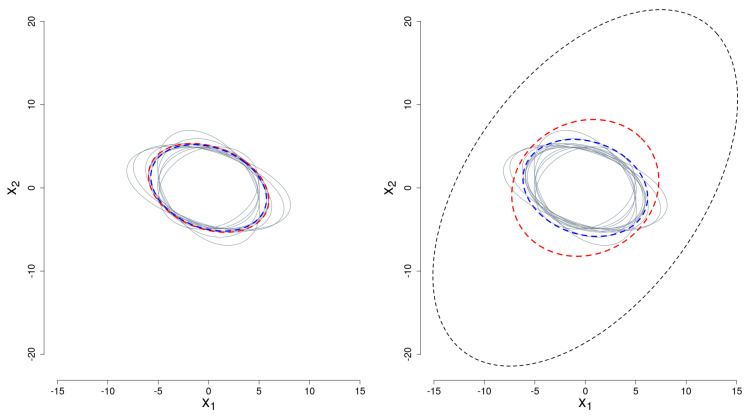
First two coordinates of ten 99th percentile ellipses (“—”). $\hat{\Sigma }$ ellipse in red (“- - -”) and $\bar{\bar{\Sigma }}$ ellipse in blue (“- - -”). Left panel: Gaussian scenario; Right panel: Gaussian scenario contaminated with anomalous covariance matrix in black (“- - -”).

In this experiment we explore the robustness of the proposed procedure in the context of detection of atypical functional data. To this aim, we generate n=100 independent sample paths from the following Gaussian stochastic model:

(14) $$\begin{array}{cc} X\left(t\right)=\xi_{1}\sin\left(t\right)+\xi_{2}\cos\left(t\right)\;\mathrm{for}\;t\in \left[0,\pi \right],\;\mathrm{and}\;\left(\begin{array}{c} \xi_{1}\\ \xi_{2} \end{array}\right) & ∼N\left[\begin{array}{cc} \mu =\left(\begin{array}{c} 0\\ 0 \end{array}\right)\;\;, & \Sigma =\left(\begin{array}{cc} 0.75 & -0.5\\ -0.5 & 0.75 \end{array}\right) \end{array}\right] \end{array}$$

that is $\left(\xi_{1},\xi_{2}\right)$ follows a zero mean bi-variate normal distribution with covariance parameters $\sigma_{11}=\sigma_{22}=0.75$ and $\sigma_{12}=\sigma_{21}=-0.5$. Using the representation techniques introduced in Section 2, we can represent these curves as points in $R^{2}$ and, moreover, we can estimate (by Maximum Likelihood) a covariance matrix $\hat{\Sigma }$ using this data representation. We replicate the previous generating process 10 times, obtaining 10 covariance matrices estimates, namely ${\hat{\Sigma }}_{i}$ for i=1,…,10. Next, we construct the *mean estimated covariance matrix* as, $\hat{\Sigma }=\sum_{i=1}^{10}{\hat{\Sigma }}_{i}/10$, and the *Karcher mean estimated covariance matrix*, $\bar{\bar{\Sigma }}\left({\hat{\Sigma }}_{1},\;\ldots ,\;{\hat{\Sigma }}_{10}\right)$. The estimations are illustrated in Figure 4-left, where each ellipse (in grey“—”) corresponds to the following equation:

$$\frac{{\left(x_{1}\cos\left({\hat{\theta }}_{i}\right)+x_{2}\sin\left({\hat{\theta }}_{i}\right)\right)}^{2}}{\sqrt{{\hat{\lambda }}_{1,i}\chi_{2,0.99}^{2}}}+\frac{{\left(x_{2}\cos\left({\hat{\theta }}_{i}\right)-x_{1}\sin\left({\hat{\theta }}_{i}\right)\right)}^{2}}{\sqrt{{\hat{\lambda }}_{2,i}\chi_{2,0.99}^{2}}}=1,\;\;\mathrm{for}\;i=1,\;\ldots ,\;10,$$

where $\chi_{2,0.99}^{2}$ is the value of a Chi-square with two degrees of freedom that accumulates 0.99 probability, ${\hat{\lambda }}_{1,i}$ and ${\hat{\lambda }}_{2,i}$ are the estimated eigenvalues, corresponding to each estimate ${\hat{\Sigma }}_{i}$, and ${\hat{\theta }}_{i}$ is the estimated rotation angle with respect to the `$x_{1}$’ axis. In addition, in the same Figure, the estimated mean $\hat{\Sigma }$ (its corresponding ellipse estimation) is shown in red (“- - -”), and in blue (“- - - ”) the Karcher mean. To introduce some anomaly in our data, in Figure 4-right, we added one ellipse constructed with an anomalous bivariate distribution with covariance matrix with elements $\sigma_{11}=\sigma_{22}=7.5$ and $\sigma_{12}=\sigma_{21}=-10$; this atypical covariance matrix corresponds to a different stochastic Gaussian model from the baseline introduced in Equation (14).

It can be observed in Figure 4-left that the average covariance matrix and the Karcher mean of the covariance matrix generate similar 99th percentile ellipses. Since the generated covariance matrices ${\hat{\Sigma }}_{i}$ are located in a small region within the cone of semi-definite positive matrices, such a region can be approximated by a linear subspace that contains the average covariance matrix. On the other hand, in Figure 4-right, the curvature of the cone is depicted by the difference in the dispersion of the anomalous covariance matrix, illustrated by the ellipse with a black-dashed line. In this scenario, the Karcher mean of the covariance matrices generates similar 99th percentile ellipse with respect to the regular scenario (left panel), which shows the robustness of the Karcher mean in the presence of outliers. Nevertheless, in the contaminated scenario (right panel), the 99th percentile ellipse generated with the simple average mean of the covariance matrices changes radically with respect to the regular scenario. The robustness in the estimation of the covariance matrix allows us to ensure that the procedure proposed in this paper, based on the estimation of the Karcher mean in the cone of positive definite matrices, will be useful when solving atypical functional data identification problems.

Last but not least, the relevant aspect of this numerical example is that, using the Karcher mean as an estimator of the center of the distribution of semi-definite positive matrices, we are minimizing the Riemannian distance, as it is defined in Section 3, and, as a consequence, the proposed method is able to identify the anomalous covariance matrix with respect to the pattern given by the rest of the distributions.

## 5. Discussion

In this work, we have explored how to combine different sources of information for anomaly detection within the framework of Entropy measures. We define entropies associated with the transformation induced by Mercer kernels, both for random variables and for data sets. We propose a new class of combination methods that generate a single Mercer kernel (that acts as a similarity measure) for anomaly detection purposes from a set of entropy measures in the context of density estimation. In particular, three combination schemes have been proposed and analysed, namely: (i) an average of the kernel matrices; (ii) the mean in the manifold that contains the kernels; and (iii) a weighting scheme that assigns the weights as the solution of an optimization problem that seeks to maximize a particular kernel entropy. Such proposals, based on the idea of building the final combined kernel matrix within the same variety where the kernel matrices to be combined live, seem to be the most successful ones on average.

An innovative application of this methodology is the use of the Karcher mean as part of a method to identify anomalous covariance matrices. The success of this proposal is due to the fact that the Karcher mean acts as an estimator of the center of the distribution of semi-definite positive matrices, while minimizing their Riemannian distance, allowing the identification of the outlying matrices with respect to the pattern given by such an estimator.

A relevant aspect for the method applicability in real problems is its complexity and costs in comparison with other alternatives. The proposals whose structure is based on a linear combination of kernel matrices have a very low computational cost based on the computation of products of constants and sums of matrices. The proposal based on the use of the Karcher mean has the typical drawback of any semidefinite programming problem, that is, the computational and memory costs are related to the size of the matrices involved. Current systems are not able to deal with dense large matrices, given that processing time and memory grow quasi-exponentially as the size of the matrices increase. See [28] for a discussion on these aspects and current trends to improve the performance of methods for the solution of semidefinite programming problems. Most applications for general dense matrices in semidefinite programming involve a few hundred data cases. Fortunately, in this particular application (outlier detection), we do not need to work with the full database to success. Due to the presence of statistical regularities, a few thousand data cases will usually be enough to collect all the relevant statistical aspects of the data set at hand.

Further research is to be afforded, especially regarding the possibility of exploring other embeddings of the data. For instance, higher dimensional transformations specific for anomaly detection could be designed. In this regard, care should be taken with the scaling of such transformations, as dimensions with large magnitudes with respect to the others may lead to suboptimal results. In this work, for multivariate data, we have compared the methodologies proposed with some multivariate outlier detection techniques. In the future, systematic experiments comparing with other well known methodologies such as XBGOD [29], LODES [30], iForest [31] or MASS [32] are to be carried out. Regarding these multivariate techniques, another interesting research line is the extension of such methodologies to functional data analysis. In this regard, suitable multivariate representations of functional data similar to those in [2] should be explored.

## Figures and Tables

**Table 1:** Percentage of TPR (sensitivity) and TNR (specificity) for synthetic multivariate data.

Experiment	$K_{G_{\sigma =0,1^{3}}}$	$K_{G_{\sigma =0,1^{2}}}$	$K_{G_{\sigma =0,1}}$	$K_{G_{\sigma =1}}$	$K_{G_{\sigma =10}}$	$K_{G_{\sigma =50}}$	$K_{G_{\sigma =100}}$	$K_{G_{\sigma =500}}$	$K_{G_{\sigma =10^{3}}}$	$K_{L}$	$K_{P}$	$\bar{K}$	$\bar{\bar{K}}$	$E_{K}$
TPR	**69.3**	**69.3**	**69.3**	65.3	0.0	0.0	0.0	0.0	0.0	**69.3**	62.6	62.6	**69.3**	62.6
TNR	**97.7**	**97.7**	**97.7**	**97.7**	92.5	92.5	92.5	92.5	92.5	**97.7**	**97.7**	**97.7**	**97.7**	**97.7**

**Table 2:** Percentage of TPR (sensitivity) and TNR (specificity) for synthetic functional data.

Experiment	Train Set (*n* = 4000)		Test Set (*n* = 2000)	
	TPR	TNR	TPR	TNR
$K_{G_{\sigma =0,1^{3}}}$	90.25	98.91	89.5	98.55
$K_{G_{\sigma =0,1^{2}}}$	90.00	98.88	90.5	98.44
$K_{G_{\sigma =0,1}}$	2.25	89.13	5.0	84.11
$K_{G_{\sigma =1}}$	0.25	88.91	0.0	81.00
$K_{G_{\sigma =10}}$	32.00	92.44	20.0	91.05
$K_{G_{\sigma =50}}$	23.75	91.52	20.0	97.77
$K_{G_{\sigma =100}}$	24.00	91.55	20.0	99.22
$K_{G_{\sigma =500}}$	14.50	90.50	44.0	57.00
$K_{G_{\sigma =10^{3}}}$	38.75	93.19	44.0	54.88
$K_{L}$	90.25	98.91	89.0	98.55
$K_{P}$	**94.75**	**99.41**	**95.5**	**99.27**
$\bar{K}$	**94.75**	**99.41**	**95.5**	**99.27**
$\bar{\bar{K}}$	38.75	93.19	44.0	55.05
$E_{K}$	**94.75**	**99.41**	**95.5**	**99.27**

**Table 3:** Summary of the data sets.

Data Set	Sample Size (*n*)	Dimension (*d*)	% of Outilers
Glass	214	9	9 (4.2%)
Vertebral	240	6	30 (12.5%)
Breast	683	9	239 (35%)
Breast (Diagnosis)	278	30	21 (5.6%)
Pima (Diabetes)	768	8	268 (35%)
Cardio	1831	21	176 (9,6%)
VDP	24	*∞* (sampled at 314 points)	35 (30.3%)
NOx	115	*∞* (sampled at 24 points)	3 (12.5%)

**Table 4:** Area under the ROC curve (AUC) for multivariate data sets.

Experiment	Glass	Vertebral	Breast Cancer	Breast Cancer (Diag.)	Pima	Cardio
$K_{G_{\sigma =0,1^{3}}}$	88.13	57.5	60.9	94.8	49.5	90.0
$K_{G_{\sigma =0,1^{2}}}$	88.51	71.4	60.8	94.7	49.7	89.5
$K_{G_{\sigma =0,1}}$	**91.49**	63.0	60.5	94.8	50.6	69.6
$K_{G_{\sigma =1}}$	82.87	66.9	62.2	94.4	71.9	65.3
$K_{G_{\sigma =10}}$	76.40	77.9	68.6	86.1	74.8	49.2
$K_{G_{\sigma =50}}$	51.49	**89.0**	68.1	85.2	48.6	46.8
$K_{G_{\sigma =100}}$	56.42	79.2	68.1	84.7	43.8	45.4
$K_{G_{\sigma =500}}$	53.90	85.8	68.1	65.2	52.4	64.8
$K_{G_{\sigma =10^{3}}}$	57.51	79.5	68.1	72.9	62.2	67.0
$K_{L}$	87.86	72.8	60.8	94.8	49.1	90.1
$K_{P}$	85.85	74.1	59.4	**96.3**	49.4	**94.8**
$\bar{K}$	85.85	66.4	62.9	94.3	48.8	**94.8**
$\bar{\bar{K}}$	88.13	82.2	60.7	94.4	**78.7**	63.4
$E_{K}$	88.13	82.2	61.4	94.4	**78.7**	**94.8**
LOF	76.8	59.3	56.4	86.9	70.9	59.6
HiCS	80.0	56.6	59.3	94.2	72.4	63.0

**Table 5:** Area under the ROC curve (AUC) for functional data sets.

Experiment	VDP	NOx
$K_{G_{\sigma =0,1^{3}}}$	**100.0**	65.7
$K_{G_{\sigma =0,1^{2}}}$	73.8	42.9
$K_{G_{\sigma =0,1}}$	88.8	48.1
$K_{G_{\sigma =1}}$	53.9	48.1
$K_{G_{\sigma =10}}$	50.7	48.1
$K_{G_{\sigma =50}}$	45.2	48.1
$K_{G_{\sigma =100}}$	52.3	48.1
$K_{G_{\sigma =500}}$	52.3	48.1
$K_{G_{\sigma =10^{3}}}$	52.3	48.1
$K_{L}$	**100.0**	57.7
$K_{P}$	**100.0**	52.0
$\bar{K}$	**100.0**	65.1
$\bar{\bar{K}}$	63.4	**70.9**
$E_{K}$	**100.0**	65.1
MBD	**100.0**	69.6
HMD	98.4	51.6
RTD	87.3	62.8
